# Correlation of 25-Hydroxyvitamin D and Serum Lipid Levels Among Patients With Type 2 Diabetes

**DOI:** 10.7759/cureus.19667

**Published:** 2021-11-17

**Authors:** Ahmed Elshebiny, Mohmmed A AlHewishel, Hussain A Al Ghadeer, Noor Alosaif, Bashayer F Al Furaikh, Muntaher S ALHejji, Hassan Ahmed A Alsahaf

**Affiliations:** 1 Internal Medicine, Diabetes and Endocrinology, Faculty of Medicine, Menoufia University, Shibin El Kom, EGY; 2 Internal Medicine, Diabetes and Endocrinology, King Faisal University, Al Hofuf, SAU; 3 Internal Medicine, King Faisal University, Al Hofuf, SAU; 4 Paediatrics, Maternity and Children Hospital, Al Ahsa, SAU; 5 College of Medicine, King Faisal University, Al Hofuf, SAU; 6 Medicine, King Faisal University, Al Hofuf, SAU; 7 Psychiatry and Behavioral Sciences, King Faisal University, Al Hofuf, SAU

**Keywords:** diabetics, saudi arabia, dyslipidemia, vitamin d, diabetes mellitus

## Abstract

Introduction

Type 2 diabetes mellitus (T2DM) is a well-known health care problem. The is a growing interest in the role of vitamin D in metabolism including glucose and lipid metabolism. This study aims to investigate the possible association between 25-hydroxyvitamin D levels and serum lipid levels among patients with T2DM.

Method

A cross-sectional study was done at the King Faisal University Health Care Center in the Eastern Region of Saudi Arabia. Ethical approval was obtained from the Ethics and Research Committee at the College of Medicine, King Faisal University. We obtained the clinical and laboratory data of patients with T2DM by searching the electronic files of patients attending the center during the period between 2014 and 2021. Data collected included age, gender, nationality, vitamin D levels, HbA1c, and lipid levels. The chi-square and independent sample t-tests were applied when appropriate, for comparisons between groups to determine significance. A P-value of less than 0.05 was considered statistically significant.

Result

The study included 191 diabetic patients, 137 (71.7%) from Saudi Arabia, and 54 (28.3%) from other countries. Patient ages ranged from 21 to 100 years with a mean age of 56.2 ± 11.8 years. Cholesterol levels were observed to be high among 61 (32.3%) patients. Considering vitamin D, the average level among male patients was 26.526 ng/ml compared to 26 ng/ml% among females (P = 0.742).

Conclusion

Further long-term and more comprehensive randomized controlled trials are needed to make a firmer conclusion and stronger evidence on this beneficial role of vitamin D treatment on T2DM.

## Introduction

Diabetes mellitus (DM) is a worldwide significant health problem [1]. DM is a heterogeneous metabolic disorder characterized by the presence of hyperglycemia due to impairment of insulin secretion, defective insulin action, or both [2]. The most common type of diabetes is type 2, affecting 90%-95% of all patients with DM [3]. Saudi Arabia holds the second-highest rate of diabetes in the Middle East (seventh highest in the world), with an approximate population of seven million living with diabetes and more than three million with pre-diabetes [4]. The prevalence of type 2 diabetes in Saudi Arabia is 32.8%. Moreover, the predicted prevalence will be 40.37% in 2025, and 45.36% in the year 2030 [5]. Furthermore, DM is consecutively associated with chronic vascular and non-vascular complications with poor general health and lower quality of life, and higher mortality rate [5]. 

Vitamin D deficiency (VDD) is defined as a serum level of 25-hydroxyvitamin D lower than 20 ng/mL [6]. VDD in Saudi Arabia is estimated to be observed in approximately 60% of the general population [7]. VDD plays a central role in several non-skeletal diseases, such as DM and cardiovascular disease [8].

Lipids abnormalities are common among patients with DM due to insulin resistance or deficiency affecting key enzymes and pathways in lipid metabolism [9]. Dyslipidemia in diabetes mellitus refers to raised low-density lipoprotein cholesterol (LDL-C), decreased high-density lipoprotein cholesterol (HDL-C) levels, or elevated triglyceride (TG) levels [10].

There is growing evidence supporting the beneficial effects of vitamin D supplementation on glycaemic control and serum lipids among patients with type 2 diabetes mellitus (T2DM). A meta-analysis was conducted from 28 randomized clinical trials studying glycaemic control in prediabetics that showed that vitamin D supplementation and improved vitamin D status improved glycaemic measures and insulin sensitivity [11]. Therefore, vitamin D supplementation can be helpful as a part of a preventive strategy for type 2 diabetes [1].

Dyslipidemia, which is common among patients with T2DM, is associated with increased cardiovascular risk [12]. As the leading cause of death and disability worldwide, cardiovascular disease (CVD) is a significant public health burden [13]. Dyslipidaemia and vitamin D deficiency are highly prevalent conditions and are independently associated with most CVD risk factors [12,14]. There is a need for more studies to figure out the exact relationship between vitamin D deficiency and serum lipids and to add more evidence about the beneficial effects of vitamin D supplementation on the prevention of CVD.

This study aims to investigate the possible association between 25-hydroxyvitamin D levels and serum lipid levels among patients with T2DM in Saudi Arabia.

## Materials and methods

Study design and participants

This is a retrospective cross-sectional study conducted at the King Faisal University Health Care Center in AlAhsa - Eastern province of Saudi Arabia to explore the correlation of 25-hydroxyvitamin D and serum lipid levels among patients with type 2 diabetes. 

Inclusion criteria

- Age more than 18 years old.

- Diagnosed with DM type II at least three months ago.

- Who is on regular follow-up with the King Faisal University Health Care Center. 

Exclusion criteria

- Age less than 18 years old.

- Recently diagnosed with DM type II.

- Patient diagnosed with DM type I.

- Hypertension, vitamin D supplementation, lipid-lowering drugs, and smoking.

- Had no coexisting disease associated with hyperlipidemia, e.g., hypothyroidism, renal disease, or hepatic disorders. 

Data collection instrument and procedure

- Patients' data were obtained by searching the electronic files of patients attending the King Faisal University's Health Care Center during the period between 2014 and 2021. 

- Data included gender, age, nationality, and blood analysis (25-hydroxyvitamin D, HbA1c levels), and lipid profile (HDL, LDL, TG, Cholesterol)).

- The sample size is equal to 191 patients who are DM type II with regular follow-up in King Faisal University's Health Care Center. 

Ethical consideration

Ethical approval was obtained from the Ethics and Research Committee in the College of Medicine, King Faisal University, IRB approval number 2020-10-62. All information was kept anonymous and maintained confidentiality. The data is used for scientific research. The data collation form by searching the electronic files.

Statistical analyses

Data were analyzed using the Statistical Package for Social Sciences (SPSS), version 21 (IBM SPSS Statistics, Armonk, NY). The chi-square and independent-sample t-tests were applied when appropriate, for comparisons between groups to determine significance. 

Data analysis

After the collection of data, it was modified, coded, and entered into statistical software IBM SPSS version 22 (SPSS, Inc. Chicago, IL). All statistical analyses were done using two-tailed tests. A P-value less than 0.05 was considered statistically significant. Descriptive analysis based on the frequency and percentage distribution was done for all categorical variables while mean, standard deviation, and range described lipid profile and vitamin D levels collectively and based on gender. Comparing lipid profile and vitamin D by patients’ gender was tested using an independent sample t-test. Additionally, comparing lipid profile and vitamin D by patients’ ages was tested using the one-way ANOVA test. Correlation analysis was used to assess the nature and strength of the unadjusted relation between HbA1c and lipid profile, while a multiple linear regression model was applied to assess the adjusted relations. The prevalence of dyslipidemia was estimated with the frequency of isolated and combined lipid abnormalities based on frequency distribution. 

## Results

In Table 1, the study included 191 diabetic patients, 137 (71.7%) from Saudi Arabia, and 54 (28.3%) from other countries. Patient ages ranged from 21 to 100 years with a mean age of 56.2 ± 11.8 years. 107 of the participants (56%) are females. 

**Table 1:** Personal characteristics of sampled patients with T2DM. T2DM: type 2 diabetes mellitus.

Personal data	No (191)	%
Country		
Saudi Arabia	137	71.7%
Developing country	50	26.2%
Developed country	4	2.1%
Age in years		
20-30	5	2.6%
31-40	9	4.7%
41-50	35	18.3%
51-60	72	37.7%
61+	70	36.6%
Gender		
Male	84	44.0%
Female	107	56.0%

Table 2 shows lipid profile and vitamin D levels among diabetic patients in Saudi Arabia. Cholesterol levels range from 102 to 300 mg/dl with a mean value of 187.3 mg/dl. Cholesterol levels were observed to be high among 61 (32.3%) patients. As for HDL, it ranged from 25 to 91 mg/dl with a mean value of 49.2 mg/dl. Moreover, 121 (66.9%) diabetic patients had high HDL level (> 40 mg/dl).Triglycerides ranged from 56.3 to 616.5 with a mean value of 147 mg /dl, and 64 (33.9%) patients had high levels. Regarding LDL, values among patients ranged from 33.5 to 195 mg/dl with a mean value of 105.2 mg/dl; 94 (57.7%) patients had high LDL levels. About vitamin D level, its value among patients ranged from 8 to 63 ng/ml with a mean value of 26.2 ±10 ng/ml. Moreover, 106 (67.5%) diabetic patients had deficient vitamin D levels, 48 (30.6%) had insufficient vitamin D levels, and three patients (1.9%) had normal levels. 

**Table 2:** Prevalence of dyslipidemia and lipid abnormalities among patients with T2DM patients. T2DM: type 2 diabetes mellitus.

Dyslipidemia	No	%
Normal	43	22.5%
Dyslipidemia	148	77.5%
Lipid profile isolated		
Normal	43	22.5%
Cholesterol (>200 mg/dl)	7	3.7%
High-density lipoprotein (<40 mg/dl)	12	6.3%
Low-density lipoprotein (>100 mg/dl)	9	4.7%
Triglyceride (>150 mg/dl)	27	14.1%
Combined		
Cholesterol & TG	21	11.0%
HDL & triglycerides	16	8.4%
Cholesterol & low-density lipoprotein & triglycerides	13	6.8%
High-density lipoprotein & triglycerides	11	5.8%
High-density lipoprotein & low-density lipoprotein & triglycerides	7	3.7%
Cholesterol & low-density lipoprotein	6	3.1%
All are abnormal	6	3.1%
Low-density lipoprotein & triglycerides	5	2.6%
Cholesterol & high-density lipoprotein & triglycerides	4	2.1%
Cholesterol & high-density lipoprotein	2	1.0%
Cholesterol & high-density lipoprotein & low-density lipoprotein	2	1.0%

Table 3 reveals the prevalence of dyslipidemia and lipid abnormalities among patients with type 2 diabetes. Dyslipidemia was detected among 148 (77.5%) patients. The most-reported isolated lipid abnormalities were elevated triglyceride levels; more than 150 mg/dl (14.1%), and low HDL; less than 40 mg/dl (6.3%). As for combined lipid abnormalities, 11% of the diabetic patients had abnormal cholesterol and triglyceride levels. Furthermore, 8.4% had abnormal HDL and LDL levels. A percentage of 6.8 had combined abnormal levels of cholesterol, LDL, and triglycerides. A percentage of 5.8 had abnormal HDL and triglyceride levels. 

**Table 3:** Lipid profile and vitamin D levels among patients with T2DM patients. T2DM: type 2 diabetes mellitus.

Lipid profile & HbA1c	No	%	Range	Mean	SD
Cholesterol level (mg/dl)					
Normal	128	67.7%	102-300	187.3	34.7
High	61	32.3%			
High-density lipoprotein level (mg/dl)					
Normal	60	33.3%	25-91	49.2	13.5
High	121	66.9%			
Triglyceride (mg/dl)					
Normal	125	66.1%	56.3-616.5	147.0	74.8
High	64	33.9%			
Low-density lipoprotein level (mg/dl)					
Normal	69	42.3%	33.5-195.0	105.2	28.7
High	94	57.7%			
Vitamin D (ng/ml)					
Deficient	106	67.5%	8.0-63.0	26.2	10.0
Insufficient	48	30.6%			
Normal	3	1.9%			

Table 4 illustrates lipid profile and vitamin D levels among diabetic patients according to their gender. Cholesterol levels were insignificantly higher among female diabetic patients than males (191.4 vs. 181.9 mg/dl; P = 0.062). HDL among females was 54.2 mg/dl compared to 42.9 mg/dl for male patients, with a statical significant difference (P = 0.001). Triglycerides and LDL levels were insignificantly different among male and female patients. Considering vitamin D, the average level among male patients was 26.526 ng/ml compared to 26 ng/ml% among females (P = 0.742). 

**Table 4:** Lipid profile and vitamin D levels among patients with T2DM patients according to their gender. T2DM: type 2 diabetes mellitus. *P-value less than 0.05.

Lipid profile & HbA1c	Gender				P-value
	Male		Female		
	Mean	SD	Mean	SD	
Cholesterol (mg/dl)	181.9	35.0	191.4	34.0	0.062
High-density lipoprotein	42.9	9.3	54.2	14.2	0.001*
Triglyceride (mg/dl)	151.3	83.8	143.7	67.3	0.580
Low-density lipoprotein (mg/dl)	106.6	32.4	104.1	25.7	0.487
Vitamin D (ng/ml)	26.5	10.5	26.0	9.7	0.742

Table 5 reveals lipid profile and vitamin D levels among diabetic patients according to age. Cholesterol levels were insignificantly higher among 40-60 aged diabetic patients than among the old (189.4 vs. 184.4 mg/dl; P = 0.627). HDL among patients aged 20-39 years was 38.0 mg/dl compared to 52.3 mg/dl for patients older than 60 years with a statical significant difference (P = 0.002). Triglycerides were significantly higher among patients aged 20-29 years old (210.6 mg/dl) than those older than 60 years (129 mg/dl) with P = 0.001. Regarding vitamin D, the average level among young patients (20-29) was 22.7 ng/ml compared to 25.5 ng/ml for patients aged 40-60 years and 28.2 ng/ml for those who are older than 60 years (P = 0.129).

**Table 5:** Correlation analysis (between vitamin D & lipid parameters) and linear regression analysis of T2DM patients showing dependency of HbA1C on other variables. T2DM: type 2 diabetes mellitus. *P-value less than 0.05.

Lipid profile	Correlation analysis		Regression analysis	
	r	P	B	P
Cholesterol (mg/dl)	-0.26*	0.001*	-0.12	0.899
High-density lipoprotein	0.04	0.654	0.03	0.829
Low-density lipoprotein (mg/dl)	-0.11	0.069	-0.04	0.673
Triglyceride (mg/dl)	-0.25*	-0.024*	-0.05	0.012*

Table 6 and the following figures clarify correlation analysis (between vitamin D & lipid parameters) and linear regression analysis of T2DM patients showing dependency of vitamin D on other variables. Vitamin D has a significant positive intermediate negative crude correlation with cholesterol level (r = -0.26; P = 0.001) and a significant negative intermediate correlation with triglycerides (r = -0.25; P = 0.024). Adjusted relation of vitamin D with lipid profile through regression model showed that only triglycerides have a significant adjusted negative relation with vitamin D keeping all other factors constant. 

**Table 6:** Lipid profile and vitamin D levels among patients T2DM patients according to their age T2DM: type 2 diabetes mellitus. *P-value less than 0.05.

Lipid profile & HbA1c	Age in years						P-value
	20-39		40-60		>60		
	Mean	SD	Mean	SD	Mean	SD	
Cholesterol (mg/dl)	185.3	33.4	189.4	35.5	184.4	33.9	0.627
High-density lipoprotein	38.0	6.9	48.7	12.4	52.3	14.8	0.002*
Low-density lipoprotein (mg/dl)	108.1	30.7	105.9	31.8	103.2	22.1	0.803
Triglyceride (mg/dl)	210.6	145.3	150.8	72.0	129.0	50.3	0.001*
Vitamin D ng/ml	22.7	8.2	25.5	9.5	28.2	10.9	0.129

## Discussion

The present study aims to explore the possible association between 25 hydroxyvitamin D levels and serum lipid levels among patients with type 2 diabetes mellitus in AlAhsa, Saudi Arabia. The majority of participants were diabetics with a Saudi nationality, equal to 137 (71.7%). We found that diabetes was more prevalent among participants aged 51 years and above (74.3% collectively). In addition, the number of female diabetic patients was slightly higher than males (56.0%, 44.0%, respectively). Keshavarz S et al. (2013) reported that the prevalence of diabetes peaked among participants aged 55-64 years old [14]. In a cross-sectional study carried out in 2016-2017 in Turaif city, Saudi Arabia, to identify the prevalence of diabetes, they found that 58.7% of the diabetic patients were aged between 36 and 65 years old, and females had a higher percentage than males (57.5%, 42.5% respectively). A significant relationship was identified between age, sex, and diabetes among the studied population (P < 0.05) in a study done by Alanazi NH et al. [15]. Mohammed MS et al. conducted a cross-sectional study to evaluate the lipid profile level in type 2 diabetes patients with vitamin D deficiency. He reported that the prevalence of DM was higher among females (63.0%) than males (37.0%) [16]. Some studies also revealed that females are more prone to have youth onset type 2 diabetes while males are more prone to have midlife onset type 2 diabetes [17]. On the other hand, Nordström A et al. found that the prevalence of type 2 diabetes was higher in older men than in older women and was related to the more significant amount of visceral fat in men [18].

In the current study, dyslipidemia was detected among 148 (77.5%) patients. High cholesterol levels were recorded among 61 (32.3%) patients. As for HDL, 121 (66.9%) diabetic patients had high HDL levels (> 40 mg/dl). Considering triglycerides, 64 (33.9%) patients had high levels, and 94 (57.7%) patients had a high LDL level. The most-reported isolated lipid abnormalities were elevated triglyceride levels more than 150 mg/dl (14.1%), and low HDL < 40 mg/dl (6.3%). The most-reported combined lipid abnormalities were abnormal cholesterol and triglyceride levels (11%), then abnormal HDL and LDL levels (8.4%), and 6.8% of the subjects had abnormal cholesterol, LDL, and triglycerides levels. A statistically significant difference was detected in HDL levels among females and males (P = 0.001). In addition, HDL and triglycerides had a statically significant difference with age groups of the diabetic patients (P = 0.002, P = 0.001, respectively).

Omotoye FE et al. conducted a study by enrolling 50 diabetic patients to evaluate the lipid profile of people with type 2 diabetes; they reported that 35 (70.0%) of diabetic patients had at least one lipid abnormality. In addition, elevated LDL-C, TCHOL, TRG, and reduced HDL-C levels were noted in 34%, 36%, 12%, and 72% of patients, respectively. Omotoye FE et al. also found that the most prevalent combined lipid abnormality was elevated LDL and reduced HDL-C. In addition, no statistically significant differences in the mean lipid profile between males and females were found. However, there was a significant difference between the mean age of males and females, and a significant difference was identified between triglycerides and blood glucose (P < 0.05) [19].

Another study was done by Kayode JA et al. (2010) to investigate the lipid profile of type 2 diabetic Nigerians. Dyslipidaemia prevalence was 50.4%. TC and HDLC (31.6%) were the most frequent lipid combination (31.6%) out of the target values in both genders, followed by decreased HDLC in 31.6% of females and 21.1% of male subjects. A statistically significant difference was observed between males and females in regards to combined lipid variables (P = 0.000). In addition, this significance was found between males and females when lipids were considered separately, TC (P = 0.000), HDL-C (P = 0.012), and LDL-C (P = 0.001) [20].

We found that 106 (67.5%) diabetic patients had deficient vitamin D levels, 48 (30.6%) had insufficient vitamin D levels. There is no statistically significant difference identified in vitamin D levels between diabetic males and females and between age groups (P = 0.742, P = 0.129, respectively). A descriptive cross-sectional study conducted among type 2 diabetic patients in Kenya to determine the prevalence of hypovitaminosis D found that vitamin D deficiency was 38.4%, and insufficiency was 21.9% among these patients [21]. In another cross-sectional study done in Saudi Arabia in 2013 for vitamin D deficiency screening among type 2 diabetic patients; suboptimal level of vitamin D was identified in the majority of the participants (98.4%), 59.8% of vitamin D deficiency, and 38.6% insufficiency. 73.6% of female participants had vitamin D deficiency, while 25.6% had vitamin D insufficiency. Among male participants, 46.9% had vitamin D deficiency, while 50.8% of them had vitamin D insufficiency. A statistically significant difference was identified between females and males (P < 0.001) [22]. A recent study done by Anyanwu AC et al. to evaluate the levels of serum vitamin D among T2DM subjects in Lagos, Nigeria found that 72 (63.2%) type 2 diabetic subjects had vitamin D deficiency in comparison to the healthy control subjects who had vitamin D deficiency; 32 (53.3%). Furthermore, no statistically significant difference between the groups was found (P = 0.44). In addition, no statistically significant difference in the distribution of vitamin D3 deficiency status by age or sex in both type 2 diabetic patients and control groups (P = 0.68, P = 0.42, respectively) [23].

This study also found a significant positive intermediate negative crude correlation was found between vitamin D and cholesterol level (P = 0.001), in addition to the significant negative intermediate correlation between vitamin D and triglycerides (P = 0.024). However, only triglycerides have a significant adjusted negative relation with vitamin D through the regression model (P = 0.012). Saedisomeolia A et al. studied the association between serum level of vitamin D and lipid profiles in type 2 diabetic patients in Iran, including 108 subjects. They revealed that serum levels of 25(OH) D had an insignificant inverse association with TG and total cholesterol (P > 0.05) and a positive correlation with HDL-C and LDL-C (P > 0.05) [24]. Mohammed MS et al. reported that there were insignificant differences in mean (cholesterol, TG, LDL-C, and HDL-C) levels in T2DM patients with vitamin D deficiency in comparison with the control group who had vitamin D level >30ng/ml (P > 0.05) [19]. Zambrana-Calví GDR et al. did another study in 2016 to investigate the changes in lipid metabolism and their relationship with vitamin D (25-OH-D) levels in type 1 DM patients under the age of 18 years. Zambrana-Calví GDR reported that some studies found that children with lower 25-OH-D levels showed higher TC, LDL-C, and TG levels, with a negative correlation seen between 25-OH-D and CT LDL-C, and apolipoprotein B. In their study, patients with a low level of 25-OH-D (<20ng/mL) had higher TG levels (P = 0.04) with a correlation was found between them (−0.230; P = 0.029) [25].

On the other hand, many studies had been done to find out the effects of vitamin D supplementation on lipid profile among T2DM patients. Ramiro-Lozano JM et al. found that after oral administration of 16,000 IU calcifediol once a week to their subjects, a significant elevation of serum levels of 25(OH) D, higher than 20 ng/ml in all cases. A statistically significant reduction was achieved in levels of total cholesterol (P = 0.04) and a non-significant trend to decrease levels of LDL cholesterol, non-HDL cholesterol, and triglycerides (P = 0.05, 0.09, 0.31, respectively). At the same time, no change was found in HDL cholesterol levels (P = 0.45) [26]. Similar results were found by Exebio JC et al.; a significant decrease in total cholesterol and triglycerides (P = 0.040, P = 0.037, respectively) was found after giving vitamin D supplement 6000 IU/day for six months. However, the effect of vitamin D supplementation can be mediated by other cofactors related to vitamin D metabolism, as the significance was lost after adjusting for confounders [27].

Glycemic control and the development of diabetes mellitus are affected by low HDL levels [28]. Mullugeta Y et al. stated that high HbA1c is associated with high triglycerides [29], while other studies revealed that DM patients with HbA1c levels exceeding 7.0% were found to have high triglycerides and LDL levels [30]. However, Klisic A et al. reported that a high HbA1c level was associated with abnormal levels of TG, TC, LDL, and HDL [31]. A parallel group of a placebo-controlled randomized pilot study showed that oral vitamin D supplementation is associated with improved glycemic control and other metabolic parameters. Moreover, significant improvement was noted in total cholesterol and LDL cholesterol levels (P = 0.05) in patients with T2DM [32]. Moreover, vitamin D deficiency is considered an additive factor in promoting atherosclerosis among T2DM patients [27,28]. Given the above, extensive studies exploring and investigating the association of vitamin D deficiency and its supplements among diabetic patients are required to identify its clinical significance for early management and prevention.

## Conclusions

Vitamin D deficiency is highly prevalent among T2DM patients. In addition, dyslipidemia among T2DM patients might be influenced by vitamin D deficiency. Further, long-term and more comprehensive randomized controlled trials are needed to make firmer conclusions and stronger evidence on this beneficial role of vitamin D treatment on T2DM.
